# Sugarcane Genotypes with Contrasting Biological Nitrogen Fixation Efficiencies Differentially Modulate Nitrogen Metabolism, Auxin Signaling, and Microorganism Perception Pathways

**DOI:** 10.3390/plants11151971

**Published:** 2022-07-29

**Authors:** Thais Louise G. Carvalho, Aline C. Rosman, Clícia Grativol, Eduardo de M. Nogueira, José Ivo Baldani, Adriana S. Hemerly

**Affiliations:** 1 Laboratório de Biologia Molecular de Plantas, Instituto de Bioquímica Médica Leopoldo de Meis, Universidade Federal do Rio de Janeiro, Rio de Janeiro 21941-901, RJ, Brazil; thaislouise@puc-rio.br (T.L.G.C.); alinecrosman@gmail.com (A.C.R.); cgrativol@uenf.br (C.G.); eduardo.nogueira@unirio.br (E.d.M.N.); 2 Laboratório de Química e Funções de Proteínas e Peptídeos, Centro de Biociências e Biotecnologia, Universidade Estadual do Norte Fluminense, Campos dos Goytacazes 28015-622, RJ, Brazil; 3 Laboratório de Genética e Bioquímica, Centro Nacional de Pesquisa de Agrobiologia, Embrapa Agrobiologia, Rio de Janeiro 23897-970, RJ, Brazil; ibaldani@cnpab.embrapa.br

**Keywords:** endophytic diazotrophic bacteria, nitrogen-fixing bacteria, differential RNA-seq transcriptome, nitrogen metabolism, plant receptors, auxin signaling

## Abstract

Sugarcane is an economically important crop that is used for the production of fuel ethanol. Diazotrophic bacteria have been isolated from sugarcane tissues, without causing visible plant anatomical changes or disease symptoms. These bacteria can be beneficial to the plant by promoting root growth and an increase in plant yield. Different rates of Biological Nitrogen Fixation (BNF) were observed in different genotypes. The aim of this work was to conduct a comprehensive molecular and physiological analysis of two model genotypes for contrasting BNF efficiency in order to unravel plant genes that are differentially regulated during a natural association with diazotrophic bacteria. A next-generation sequencing of RNA samples from the genotypes SP70-1143 (high-BNF) and Chunee (low-BNF) was performed. A differential transcriptome analysis showed that several pathways were differentially regulated among the two BNF-contrasting genotypes, including nitrogen metabolism, hormone regulation and bacteria recognition. Physiological analyses, such as nitrogenase and GS activity quantification, bacterial colonization, auxin response and root architecture evaluation, supported the transcriptome expression analyses. The differences observed between the genotypes may explain, at least in part, the differences in BNF contributions. Some of the identified genes might be involved in key regulatory processes for a beneficial association and could be further used as tools for obtaining more efficient BNF genotypes.

## 1. Introduction

Sugarcane (*Saccharum* spp.) is an important crop worldwide, being a major source for the production of sugar and renewable energy sources such as ethanol. It is characterized by a highly complex polyploid genome, and the cultivated commercial varieties are interspecies hybrids derived mainly from crosses between the sucrose-accumulating *Saccharum officinarum* L. (2n = 8x = 80) and the disease-resistant but low-sucrose-containing *Saccharum spontaneum* L. (2n = 5x to 12x = 40–128) [1]. Therefore, genetic regulators and physiological processes can differ greatly between sugarcane varieties. Studies on sugarcane have shown that distinct sugarcane genotypes present different responses to diazotrophic bacteria association. The SP70-1143 (SP) variety may obtain up to 72% of their nitrogen needs from BNF, while the Chunee (CH) genotype can only obtain around 32% of their nitrogen from BNF [2,3]. During the Brazilian sugarcane crop history, commercial hybrids were selected for high yields with low inputs of inorganic nitrogen fertilizer [4,5,6,7]. This strategy might have led to the assortment of cultivars with higher contributions of Biological Nitrogen Fixation (BNF), the ability to grow with low nitrogen fertilizer inputs. Several Brazilian sugarcane cultivars with significant contributions of N_2_ fixation have been identified, as they can obtain up to 72% of the plant nitrogen (N) requirements and over 150 Kg N fixed ha^−1^ per year [2,4,8,9]. Efficient BNF has a positive impact on Brazilian agriculture, as sugarcane cultivation applies one third to one fifth of the amount of N fertilizer used in other countries [3,10].

Different species of nitrogen-fixing bacteria isolated from sugarcane tissues might be contributing to the quantified BNF rates, such as *Gluconacetobacter diazotrophicus*, *Herbaspirillum seropedicae*, *Herbaspirillum rubrisubalbicans*, *Azospirillum brasilense* and others [6,11]. The diazotrophic bacteria are classified as associative and/or endophytic, as they colonize root surfaces or intercellular spaces and vascular tissues of most plant organs, without causing visible plant anatomical changes or disease symptoms [12,13,14]. Furthermore, the plant–bacteria association can be beneficial to sugarcane development, by promoting root growth and an increase in plant biomass and productivity [15,16]. Bio-inoculants formulated with nitrogen-fixing bacterial species have been successfully used in Brazilian agriculture; nevertheless, the increase in sugarcane biomass and yield is dependent on soil conditions and plant genotype. Distinct sugarcane genotypes have different rates of BNF, and plant genetic factors might regulate the efficiency of the process [2,3,13,17]. An increasing amount of data on plant genes and physiological pathways that are responsive to the endophytic and associative bacteria in non-leguminous plants have been generated in recent years [18,19,20,21,22,23].

An intriguing question that remains to be elucidated is how the different plant genotypes perceive environmental and endophytic nitrogen-fixing bacterial signals to activate proper responses that will finally culminate, or not, in physiological adaptations that are beneficial to the plant. To address that, in this work a differential transcriptome approach was used to unravel plant genes that are differentially regulated during a natural association with diazotrophic bacteria in germinated root and shoot stalks from two sugarcane genotypes that are considered models for contrasting BNF efficiency studies. To discriminate intrinsic differences among the genotypes, RNA-seq data of diazotrophic-free plants, grown in hydroponics, were also generated. First, a new sugarcane reference transcriptome database was generated, specific to two sugarcane genotypes that have different contributions from BNF. A differential expression analysis revealed several plant physiological processes that are differentially regulated between the BNF-contrasting genotypes. Some gene expression profiles were intrinsic of each genotype, even in the absence of the endophytic nitrogen-fixing bacteria. In parallel, similar expression profiles of genes observed in naturally colonized stalks were validated in plants inoculated with diazotrophic bacteria, indicating that they may participate in the regulation of the association with diazotrophic bacteria. Further physiological and biochemical analyses functionally supported the biological involvement of microorganism recognition (plant receptor), nitrogen metabolism and auxin signaling pathways in the differences among the two contrasting BNF genotypes. Altogether, the data indicated that some differences among the contrasting BNF genotypes were genetically intrinsic of each genotype; however, the efficiency of the association with endophytic diazotrophic bacteria could explain, at least in part, the distinctions in BNF and growth promotion between the contrasting genotypes. The discovery of these features could help the management of crops in order to achieve higher levels of productivity.

## 2. Results and Discussion

### 2.1. RNA Sequencing of Sugarcane Genotypes with High and Low BNF Efficiencies

To unravel plant genes that are differentially regulated in two sugarcane BNF-contrasting genotypes, a differential transcriptome approach was used. Two sugarcane genotypes that have been considered as models of contrasting BNF efficiencies were characterized: the commercial cv. SP70-1143, which could obtain up to 72% of its N requirements from BNF, and the wild non-commercial species *Saccharum barberi* (Chunee), which obtains low rates of N fixed by BNF [2]. In the field, sugarcane is mainly propagated vegetatively, through the germination of roots and shoots from secondary meristems at internodes of the stalks. After the emergence of stalk roots and shoots, around 15 days after planting, a high increase in diazotrophic bacterial numbers occurs that might trigger various plant responses to assure the establishment of an efficient and beneficial association with the novel plant microbiome. Therefore, root and shoot tissues of stalks of the BNF-contrasting genotypes, at the early developmental phase (15 days after planting), were used to construct RNA-seq libraries. To distinguish the differential gene expression that is under genotypic control, rather than influenced by the colonization by diazotrophic bacteria, plants grown in hydroponic culture derived from in vitro cultured plantlets free of diazotrophic bacteria were also used for the construction of RNA-seq libraries. In total, 16 libraries of 8 different plant materials were constructed and sequenced (Table S1). From each library, between 2 to 14 million reads were sequenced, and the data sequencing of all libraries represented a total of 110,478,905 reads (Table S1).

**Figure 1:**
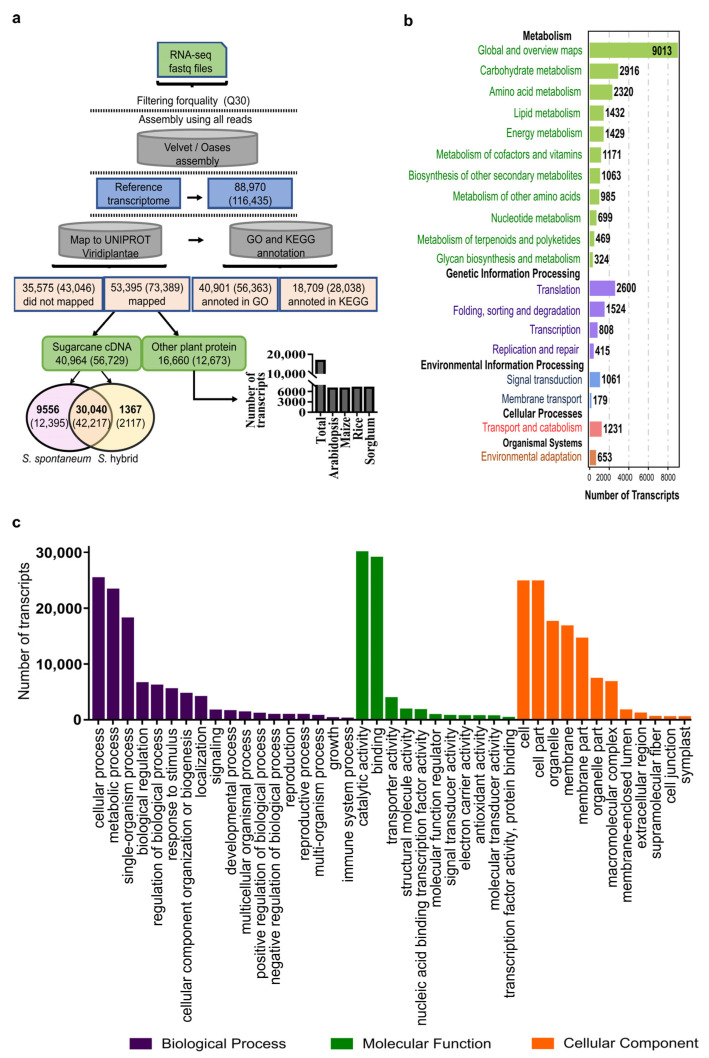
Pipeline of the construction of the sugarcane reference transcriptome. (**a**) A total of 16 libraries were analyzed and sequencing results were used to construct a sugarcane reference transcriptome. Over 130 million reads were used for Velvet-Oases pipeline assembly that resulted in 88,970 loci and 116,435 transcripts. A total of 53,395 loci mapped with plant sequence databases and, within these, 40,964 matched with sugarcane genome sequences available in public databases. A group of 12,673 loci mapped with other plant species databases. A group of 35,575 loci did not map within Viridiplantae databases. The figure also shows the number of sugarcane transcripts with no matches in sugarcane ESTs database that presented homologues in Arabidopsis, maize, rice and sorghum with an e-value cut-off < 10^−5^, over 70% of identity and best hits. The transcripts were categorized in (**b**) KEGG pathway annotation and (**c**) GO database, using OmicShare tools. Only the most represented classes in sugarcane reference transcriptome were displayed.

First, since public DNA sequences are not available for these two BNF-contrasting genotypes, a sugarcane reference transcriptome suitable to this study was generated. A strategy of de novo contigs assembly was applied (Figure 1a) yielding a total of 116,435 unique transcripts that corresponded to 88,970 unique loci. This dataset with the reference transcriptome was named RT1. The transcript sequences presented an average length of 699 bp, a total length of approximately 81,456 Mb and an N50 of 1405 bp. The sugarcane transcriptome sequences were evaluated against proteins of the Viridiplantae UNIPROT database (https://www.uniprot.org/uniprot/?query=viridiplantae&sort=score, accessed on 15 November 2020) using the BLASTX and an e-value cut-off threshold of ≤ 1 × 10^−5^. A total of 53,395 loci (73,389 transcripts) were mapped with plant protein sequences (Figure 1a) representing 60% of the RT1 loci (63% of the transcripts).

Likewise, the conservation of RT1 sequences was analyzed with proteome databases of Arabidopsis, rice, maize and sorghum using the BLASTX tool (Table 1). The largest number of RT1 loci (81.87%) showed significant similarity with sorghum proteins followed by maize (79.47%) and rice (76.48%). As expected, a lower number of RT1 loci showed significant similarity with the model dicot Arabidopsis (64.83%) when compared with monocots (76–81%). To assign putative functional categories, the sugarcane RT1 transcripts were classified based on Gene Ontology (GO) and the Kyoto Encyclopedia of Genes and Genomes (KEGG) analysis. A total of 40,901 loci and 56,363 transcripts were functionally categorized and putative orthologs of genes involved in various pathways and cellular processes were found to be conserved in sugarcane (Figure 1b,c).

The Viridiplantae transcripts of RT1 were mapped against the sugarcane genome sequences available in public databases, using the BLASTN tool with an e-value cut-off threshold of ≤ 1 × 10^−5^. A total of 40,964 loci (56,729 transcripts) matched with sugarcane genome sequences (Figure 1a). A group of 12,673 loci (16,660 transcripts) that were not mapped on the sugarcane databases were mapped with cDNA data of other plant species and could represent sugarcane sequences that were not identified in other available sugarcane sequence databases. Most of these transcripts showed a significant hit with the protein of Arabidopsis, rice, sorghum and maize (Figure 1a). Among the novel sugarcane transcripts, the five most representative functionals in the KEGG class B categories were the global and overview maps, carbohydrate metabolism, translation, amino acid metabolism and folding, and sorting and degradation (Figure 1b). In addition, the transcripts were also annotated in several GOs divided into three groups: biological process (BP), molecular function (MF) and cellular component (CC), as shown in Figure 1c. The most represented GOs were catalytic activity (MF), binding (MF), cellular process (BP), metabolic process (BP), and cell (CC). There is also a group of 35,575 loci (43,046 transcripts) that did not map with any plant databases (Figure 1a).

### 2.2. Differential Expression Profiles in the Two Sugarcane BNF-Contrasting Genotypes

Differential gene expression profiles between the sugarcane BNF-contrasting genotypes were generated to identify genes and metabolic pathways that might determine the establishment of an efficient BNF. A summary of the reads and transcripts mapped in each generated library is presented in Table S1.

**Figure 2:**
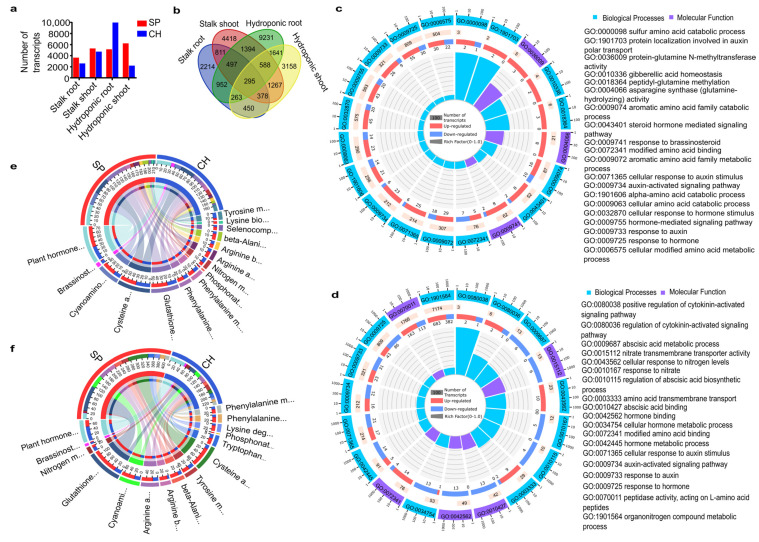
Quantitative evaluation of differentially expressed transcripts between BNF-contrasting genotypes. (**a**) The figure shows the number of differentially expressed transcripts that are more represented in each genotype for each condition. (**b**) Venn diagram shows the number of transcripts differentially expressed between SP and CH in each set (tissue and growth conditions). (**c**,**d**) Gene ontology (GO) enrichment analysis of DET in (**c**) stalk root and (**d**) stalk shoot. The first circle: the most enriched terms; outside the circle is the coordinated ruler of the number of transcripts. The second circle: the classification number in the background. The more transcripts, the longer the bar. The third circle: bar graph of the ratio of the DETs; red represents the ratio of the upregulated transcripts in SP, and blue represents it in CH. The fourth circle: The Rich Factor value of each category (the number of foreground transcripts in the category divided by the number of background transcripts). (**e**,**f**) The circus diagrams represent the KEGG pathway enrichment analysis of DETs in stalk root (**e**) and stalk shoot (**f**) of SP and CH. The figures show only the most significantly enriched GO terms and KEGG pathways that are involved in hormone, nitrogen, and amino acid pathways, such as: plant hormone—plant hormone signal transduction; Brassinost—Brassinosteroid biosynthesis; Nitrogen m—Nitrogen metabolism; Glutathione—Glutathione metabolism; Cyanoami—Cyanoamino acid metabolism; Arginine a—Arginine and proline metabolism; Arginine b—Arginine biosynthesis; beta-Alani—beta-Alanine metabolism; Tyrosine m—Tyrosine metabolism; Cysteine a—Cysteine and methionine metabolism; Tryptophan—Tryptophan metabolism; Phosphonat—Phosphonate and phosphinate metabolism; Lysine deg—Lysine degradation; Phenylalanine—Phenylalanine, tyrosine and tryptophan biosynthesis; Phenylalanine m—Phenylalanine metabolism; Selenocomp—Selenocompound metabolism; Lysine bio—Lysine biosynthesis. Red represents the number of the upregulated transcripts in SP, and blue in CH. The GO and KEGG enrichment analyses were performed using OmicShare tools.

A total of 38,409 transcripts were identified as differentially expressed between SP and CH in all of the analyzed samples (Figure 2a). Among them, 721 transcripts were found to be expressed only in one of the genotypes. A list of comparisons and the datasets of differentially expressed transcripts (DETs) are presented in Table S2. Among the different pairwise comparisons between genotypes, some DETs were specific to tissues and growth conditions. A group of 2214 transcripts were only differentially expressed between SP and CH in samples of roots of germinated stalks, while 4418 transcripts were specifically regulated between SP and CH only in samples of shoots of germinated stalks (Figure 2b). A set of 9231 and 3158 transcripts were specifically regulated between SP and CH in samples of roots and shoots of plants grown in hydroponics, respectively (Figure 2b). As shown in Figure 2a,b, the number of transcripts that were more expressed in SP is greater than those in CH, except for DETs from the roots of the hydroponic culture.

To evaluate the plant processes that are differentially regulated in the BNF-contrasting genotypes naturally colonized with diazotrophic bacteria, functional categories in the different dataset comparisons were analyzed. In this way, all DETs were subjected to GO and KEGG enrichment analysis, which were performed using OmicShare tools (www.omicshare.com/tools, acessed on 15 November 2020). Several GO terms were significantly enriched (Table S3), including those involved with nitrogen and hormone metabolism in roots (Figure 2c) and in shoots (Figure 2d) that were selected as a sub-bank for the transcriptome analysis, as diazotrophic bacteria are mainly known for their ability to fix atmospheric N and to produce phytohormones. Remarkably, the KEGG enrichment analysis showed several significantly enriched pathways (Figure S1 and Table S4), and a greater number of transcripts in plant hormone signal transduction, as well as nitrogen and amino acid metabolism—three physiological processes that are closely related to plant association with nitrogen-fixing bacteria—were observed in roots (Figure 2e) and in shoots (Figure 2f). In addition, most of these genes involved in these specific pathways were upregulated in SP in comparison with CH. Gene families involved in cell wall biosynthesis pathways that were differentially expressed between SP and CH were recently reported by analyses of the same RNA-seq database generated in the present work [23]. The data show that the gene expression profile is quite different between the two sugarcane genotypes naturally colonized with the diazotrophic bacteria, suggesting that regulation of some pathways in SP and CH might be participating in the control of BNF rates. Some of the pathways will be discussed in more detail in a further topic.

**Figure 3:**
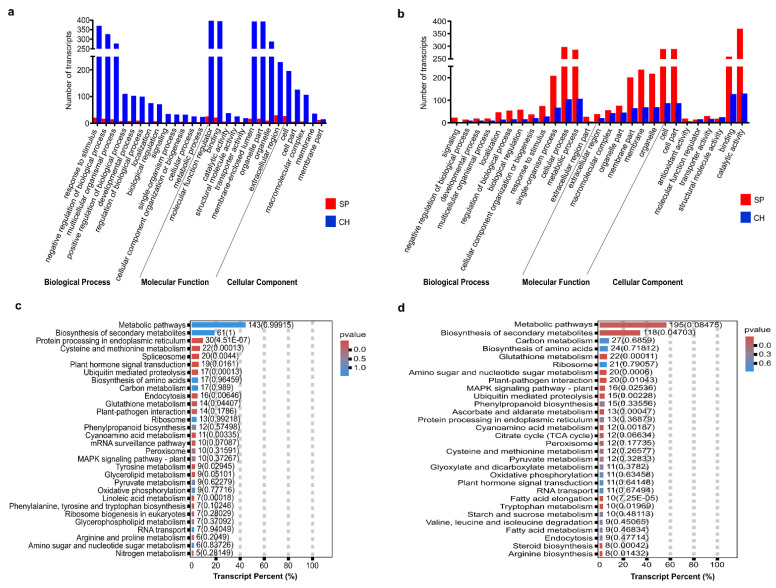
Analysis of DETs between BNF-contrasting genotypes that show a common pattern of regulation in stalks and hydroponics. (**a**,**b**) The figures show the number of differentially expressed transcripts that are more represented in GO categories. Red represents the number of the upregulated transcripts in SP, and blue in CH. (**a**) shows data of roots and (**b**) shows data of shoots. (**c**,**d**) The graphs show the KEGG enrichment analysis (**c**) in roots and (**d**) in shoots. Only the most represented classes in the sugarcane reference transcriptome were displayed. KEGG enrichment analysis of DETs was carried out through OmicShare tools.

**Figure 4:**
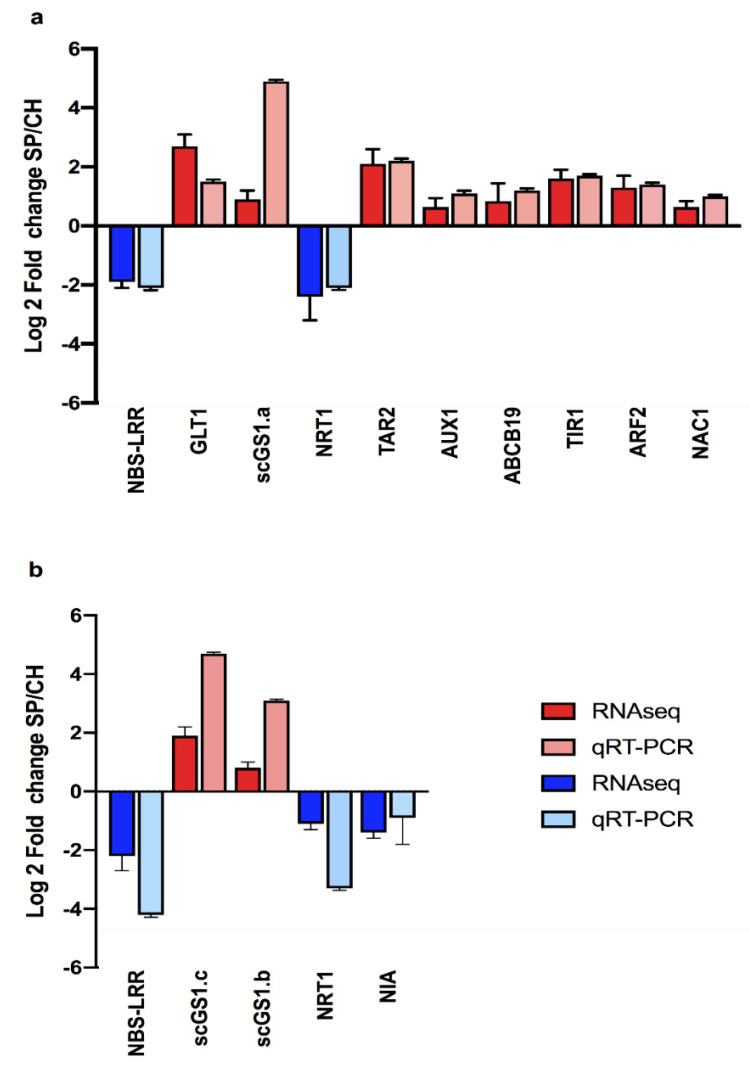
Validation of RNA-seq results by qRT-PCR analysis. Patterns of expression of genes were analyzed in roots (**a**) and (**b**) shoots of the two contrasting BNF-genotypes. Comparison of the pattern of expression of DETs between SP and CH in stalk tissues, showing log2 values in the transcriptome libraries and the qRT-PCR analyses. For the expression in the transcriptome libraries, results are presented as the ratio SP/CH of the RPKM of each transcript. Bars represent mean ± standard deviation of the relative mRNA expression in two biological replicates. For the qRT-PCR data, results are presented as the ratio of expression of each transcript (relative to GAPDH and 28S rRNA) in each sample. Bars represent mean ± standard deviation of the relative mRNA expression in three biological replicates (three plants) and each biological replicate analyzed with three technical replicates.

Some identified DETs could be related to the BNF differences observed between SP and CH, and others could reflect intrinsic differences among the genotypes, possibly not related with the diazotrophic bacteria colonization. To address that, DETs from stalk datasets (naturally colonized with diazotrophic bacteria) were searched in the datasets of plants grown in hydroponics (diazotrophic-free). A group of 2422 transcripts—1138 transcripts in roots and 1284 transcripts in shoots—presented the same pattern of differential expression between SP and CH in the stalks and hydroponics. Their functional categorization in GO and KEGG are presented in Figure 3. A higher number of upregulated DETs in CH was observed in the roots (Figure 3a). In contrast, in the shoots, a greater number of upregulated DETs was observed in SP (Figure 3b). Among the DETs analyzed, there were transcripts involved in hormone response, nitrogen and amino acid metabolism (Figure 3c,d). The differential expression of two of them (NBS and NRT1), both in stalk and hydroponics tissues, were validated by qRT-PCR (Figure 4a and Figure S1).

As shown in Figure 4, Figures S1 and S2, the RNA-seq data were validated by qRT-PCR for all transcripts analyzed in roots and shoots. Ten of these validated transcripts are among the 721 exclusive transcripts of each genotype (Figure S2). Other transcripts are members of plant–bacteria recognition (1), nitrogen signaling/metabolism (6) and plant hormone response (6) (Figure 4). These results will be discussed later in the article in each appropriate section.

Next, to better understand the physiological and molecular differences between the two BNF-contrasting genotypes, plant pathways that might be critical for the establishment of a successful endophytic association were analyzed in more detail. These analyses focused on mechanisms that could be closely involved in plant responses to a beneficial association with the nitrogen-fixing bacteria, such as nitrogen metabolism, auxin signaling pathways, and plant recognition of the beneficial bacteria.

### 2.3. Plant Receptors Involved in Plant–Bacteria Recognition

**Figure 5:**
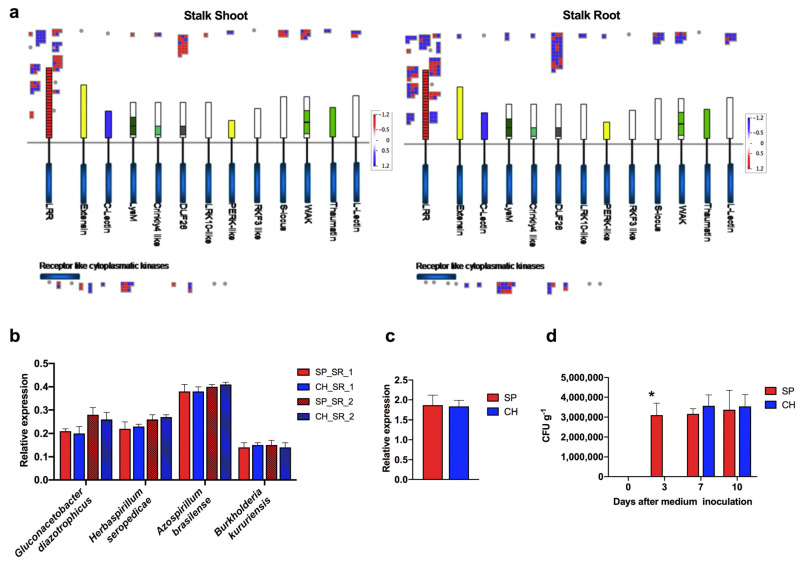
Evaluation of biotic stress response pathways differentially regulated between BNF-contrasting genotypes and bacteria colonization. (**a**) Schematic representation of different plant receptor families, showing the DETs identified in shoot and root tissues of germinated stalks. In red are represented the transcripts that are more expressed in SP and in blue are represented the ones more expressed in CH. (**b**) Relative levels of *G. diazotrophicus*, *H. seropedicae*, *A. brasilense* and *B. kururiensis* colonizing sugarcane were measured by qRT-PCR of bacterial 23S rRNA in germinated stalks 15 days after planting. Bacterial 23S rRNA levels were normalized with plant 28S rRNA and GAPDH levels. Bars represent mean ± standard deviation of the relative mRNA expression in three biological replicates (three plants) and each biological replicate analyzed with three technical replicates. (**c**) Relative levels of *G. diazotrophicus* colonizing sugarcane were measured by qRT-PCR of bacterial 23S rRNA in hydroponic cultured plants 10 days after inoculation with *G. diazotrophicus*. Bacterial 23S rRNA levels were normalized with plant 28S rRNA and GAPDH levels. Bars represent mean ± standard deviation of the relative mRNA expression in two biological replicates (three plants) and each biological replicate analyzed with three technical replicates. (**d**) Quantification of bacterial numbers of SP and CH plants grown in hydroponic culture and inoculated with *G. diazotrophicus* using MPN assay. Analyses were performed 0, 3, 7 and 10 days after inoculation. Bars represent mean ± standard error of the relative mRNA expression in three biological replicates (three plants) and each biological replicate analyzed with three technical replicates. Asterisk marks statistical significance between SP and CH (* *p* < 0.05), performed by statistical t-test.

BNF beneficial features depend on bacteria establishment in plant tissues that might be controlled by plant–bacteria recognition pathways that operate during the early stages of colonization. The bacteria adhesion to the cell wall of the root surface cells might trigger the appropriate recognition processes to establish a beneficial association [14,24,25,26,27]. As beneficial and pathogenic bacteria share several features of recognition, this process must be fine-tuned for a proper response. Key regulators of these mechanisms are plant receptors [28]. Therefore, plant receptor families involved in plant–bacteria recognition were compared between the BNF-contrasting genotypes naturally colonized with diazotrophic bacteria. A high number of DETs involved in bacteria recognition (68 in stalk roots and 67 in stalk shoots), i.e., plant receptors, were identified (Figure 5a and Table S5). A total of 33 and 23 DETs were upregulated in the SP root and shoot, respectively, while 25 and 44 DETs were downregulated in the SP root and shoot, respectively. The DETs belonged to different families of plant receptors and are represented in Figure 5a. The functional annotation identified several members of Receptor Like Kinases (RLK) families, such as Leucine Rich Repeat containing Receptor Like Kinases (LRR-RLKs), Wall Associated Kinases (WAKs), Lectin Receptor Like Kinases (LecRLKs) and Lys-motif receptors (LysMs) as differentially expressed between SP and CH.

#### 2.3.1. NBS-LRR Proteins

Fifty-nine DETs within biotic stress members belonged to the family of LRR proteins. This family of proteins is responsible for the first interaction of plants with pathogens, regulating the activation of defense responses [29,30]. Many plant resistant genes (R genes) encode NBS-LRR proteins (nucleotide-binding/leucine-rich repeat domains) [31]. Three DETs were annotated as NBS-LRR-kinase proteins (one for stalk roots and two for stalk shoots) (Table S5) and they showed higher expression levels in CH roots and shoots (Figure 5a and Table S5). The RNA-seq differential expression analysis data of one member of the NBS-LRR family were confirmed in the qRT-PCR analysis (Figure 4a and Table S5). Remarkably, two of the transcripts encoding NBS-LRR were more expressed both in the hydroponic and stalk root of CH than of SP; qRT-PCR validation was performed for one of them (Figure 4a and Figure S1). The data suggest that this expression profile is under genotype control, independent of the diazotrophic bacteria colonization, and could possibly have a role in the establishment of an efficient association with the diazotrophic bacteria.

#### 2.3.2. LRR-Kinase Family

Most differentially expressed plant receptors belong to the LRR-kinase family. In this receptor family, 23 and 10 DETs were upregulated in SP stalk roots and shoots, respectively; and 10 and 16 DETs were downregulated in SP stalk roots and shoots, respectively (Figure 5a and Table S5). Among the LRR-kinase proteins, SHR5 is a LRR-RLK that was previously identified as specifically repressed in sugarcane inoculated with beneficial diazotrophic bacteria such as *G. diazotrophicus*, *Herbaspirillum* spp. and *A. brasilense* [22]. As observed for the three NBS-LRR transcripts, SHR5 was more expressed in CH than in SP. On the other hand, 303 ESTs encoding putative LRR-RLKs were induced in sugarcane inoculated with *G. diazotrophicus* and *H. rubrisubalbicans* [32].

The LRR receptor kinase flagellin-sensitive 2 (FLS2), that acts as a pattern-recognition receptor for the bacterial PAMPs flagellin and contributes to resistance against bacterial pathogens [33], was more expressed in the CH stalk shoot samples. Besides its role in pathogen perception, the FLS2 receptor could also be involved in beneficial association signaling. In Arabidopsis and *Vitis vinifera*, Flagellin-Sensitive 2 (FLS2) LRR-RLK that recognizes and directly binds flg22, the immunogenic epitope of the PAMP flagellin, was transcriptionally induced in plants inoculated with the plant-growth-promoting bacteria (PGPB) *Burkholderia phytofirmans* [34].

Altogether, the results suggest that the genotype with a higher efficiency of association with diazotrophic bacteria, leading to higher rates of BNF, has lower levels of expression of members of the LRR receptor family involved in plant defense against pathogens.

#### 2.3.3. Endophytic Bacterial Colonization

Our data demonstrated the regulation of plant receptors known to be involved in plant–bacteria recognition. NBS-LRR, FLS2, WAK as well as SHR5 were more expressed in CH than in SP, and as observed for other models, this regulation could be important for the proper recognition of diazotrophic bacteria and the establishment of a beneficial association. An important question to be addressed is if the differences in BNF efficiency are, in part, a consequence of the efficiency of bacterial colonization inside plant tissues, which could be controlled by specific plant recognition and/or defense genes. To address that, bacterial colonization was quantified in the two BNF-contrasting genotypes by qRT-PCR of diazotrophic bacterial ribosomal RNA, in terms of total RNA from stalk roots and shoots. Primers amplifying specific regions of 23S or 16S rRNA constitutive genes were used to identify *G. diazotrophicus*, *H. seropedicae*, *A. brasilense* and *Burkholderia kururiensis*. This strategy is widely used to detect microorganisms in plant tissues and in phylogenetic studies [35,36,37,38]. No differences in bacterial colonization were observed between the SP and CH samples for any of the four bacterial rRNA relative expressions analyzed (Figure 5b). The result indicates that diazotrophic bacterial numbers might not determine the differences in BNF efficiencies among genotypes.

Next, to test if the physiological status of beneficial diazotrophic bacteria could be differentially modulated when colonizing the contrasting genotypes, the growth dynamic of diazotrophic bacteria recovered from SP and CH plants inoculated with *G. diazotrophicus* was evaluated. Hydroponically grown plants, free of diazotrophic bacteria, were inoculated with *G. diazotrophicus*, and bacterial colonization was quantified by qRT-PCR (Figure 5c) and MPN (Figure 5d). The qRT-PCR analysis revealed similar levels of colonization by *G. diazotrophicus* in SP and CH (Figure 5c), supporting the data from naturally colonized germinated stalks. For MPN analysis, seven and ten days after semi-solid medium growth, the numbers of diazotrophic bacteria recovered from SP and CH were similar (Figure 5d), corroborating the results of qRT-PCR (Figure 5c). However, three days after growth in semi-solid medium, there was a significant difference between bacterial numbers recovered from plant tissues of contrasting genotypes: while SP showed an average of 3 × 10^6^ CFU g^−1^ of *G. diazotrophicus*, this bacterium could not be recovered from CH tissues (Figure 5d). The longer time necessary for *G. diazotrophicus* to be recovered from CH plant tissues could suggest a difference in the physiological status of diazotrophic bacteria colonizing SP and CH.

In addition to the rates of bacterial colonization in plant tissues, bacteria metabolism might also be important for the beneficial effects to plants. The growth delay of diazotrophic bacteria recovered from CH tissues could imply that the two contrasting genotypes might offer a distinct environment for bacterial growth, as suggested by the differences in several metabolic and defense pathways found in the transcriptome analyses. As a consequence, the diazotrophic bacteria could stay in a lower active physiological status when colonizing CH, compared with SP colonization. This could account, at least in part, for the lower BNF contributions of the CH genotype. In addition, the activation of defense pathways could suggest that in an inefficient sugarcane–diazotrophic bacteria association, the plant seems to fail to recognize the bacteria as beneficial, but rather identifies it as a pathogen. These data may suggest that an important control of the efficiency of the association, regulated by both the genotype and the environment, is already set in the early stage of plant–bacterium recognition.

### 2.4. Plant N Assimilation and Metabolism and Amino Acid Metabolism Pathways

**Figure 6:**
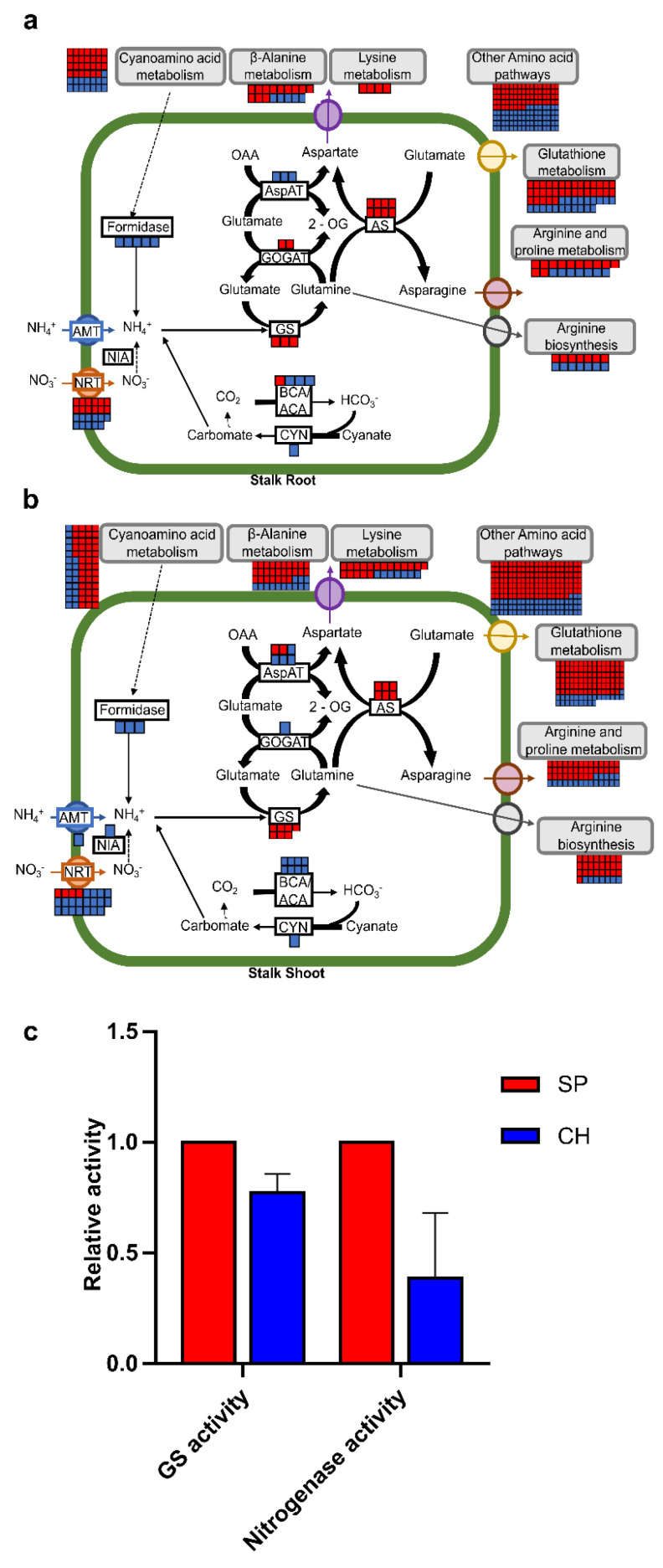
Evaluation of nitrogen and amino acid metabolism pathways differentially regulated between BNF-contrasting genotypes. Schematic representation of N assimilation and transport, showing the DETs identified in root (**a**) and shoot (**b**) tissues of germinated stalks. In red are represented the transcripts that are more expressed in SP and in blue are represented the ones more expressed in CH. (**c**) Enzyme activity of GS and nitrogenase was evaluated in 15-day germinated stalks and is represented as relative activity in relation to SP70-1143 values. Bars represent mean ± standard deviation of five biological replicates. Asterisks (*) represent significant differences between SP and CH through t-test (*p* < 0.05). GOGAT—glutamate synthase; GS—glutamine synthetase; AS—asparagine synthetase; NIA—nitrate reductase; NRT—nitrate transporter protein; AMT—ammonium transporter; 2-OG—2-oxoglutarate; Gln—glutamine; Glu—glutamate; Asn—asparagine; NO_3_^−^—nitrate; NH_4_^+^—ammonium; BCA—beta carbonic anhydrase; ACA—alfa carbonic anhydrase; AspAT—aspartate aminotransferase; OAA—oxaloacetate.

Much evidence suggests that endophytic diazotrophic bacteria can contribute fixed N to the plant or can change plant nitrogen metabolism [39]. Therefore, N-assimilation pathways were compared between the BNF-contrasting genotypes naturally colonized with diazotrophic bacteria. A total of 99 transcripts related to N assimilation and metabolism were identified as differentially expressed in the contrasting genotypes: 48 DET were found in roots and 51 in shoots (Figure 6a,b and Table S6). In addition, several transcripts categorized in amino acid metabolism were identified, 468 DET were found in roots and 275 in shoots (Figure 6a,b and Table S6).

#### 2.4.1. Nitrate Transporters

The nitrate (NO_3_^−^) transporters (NRTs) were one of the most represented functional categories related to nitrogen metabolism that were differentially expressed during beneficial associations. A total of 46 DETs were found in the database (Figure 6a,b and Table S6). In plants, nitrate can be absorbed and translocated through transmembrane proteins, which mainly consist of NITRATE TRANSPORTER 1 (NRT1)/PEPTIDE TRANSPORTER (PTR) family (NPF) and NRT2 [40,41]. Besides nitrate, the NPF family transports several components as substrates, including dipeptides, glucosinolate and phytohormones [42,43]. By contrast, NRT2 members have only been reported to transport nitrate [42].

SP showed six DETs that were more expressed and annotated as the NPF8 subfamily (NPF8.1/PTR1 and NPF8.3/PTR2)—four in the shoot and two in the root—which in Arabidopsis encodes dipeptide transporters [44], as well as two DETs in the shoot annotated as NPF2.11, which encodes a glucosinolate transporter, the most important secondary metabolite for plant defense [45]. In addition, two DETs annotated as the NPF2 subfamily (NPF2.3 and NPF2.6), which encodes a member of the NAXT, three DETs annotated as NPF3.1, which encodes nitrate or peptide transporters, one DET annotated as NPF4.3, which encodes an ABA transporter, and two DETs annotated as the NPF6 subfamily (NPF6.3/NRT1.1 and NPF6.4), which encodes nitrate transporters, were more expressed in stalk roots of SP.

On the other hand, the CH genotype showed a greater number of more expressed transcripts (30 DETs) that encode members of the nitrate transporter family, mainly in shoot tissues (19 DETs) (Figure 6b and Table S6). In CH shoots, the annotated DETs and the number of transcripts were: NPF2.11 (5); NPF3.1 (1); NRT3.2 (1) that codifies a high-affinity nitrate transporter [46]. NPF4.5 and NPF4.6, from the NPF4 subfamily (4), which are known as ABA transporters [47]; NPF5.1–3, from the NPF5 family (1), which encode dipeptides/ABA influx transporters [48,49]; NPF6.3 and NPF6.4, from the NPF6 subfamily (6); NPF7.3 (1) that encodes a nitrate transporter in the xylem [50]; NPF8.1/PTR1, NPF8.2/PTR5 and NPF8.3/PTR2, from the NPF8 subfamily (2). In CH roots, the annotated DETs and the number of transcripts were: NPF4.5 and NPF4.6, from the NPF4 subfamily (2); NPF5.1–3, from the NPF5 subfamily (2); NPF8.1/PTR1, NPF8.2/PTR5 and NPF8.3/PTR2, from the NPF8 subfamily (4). Moreover, seven DETs codifying for NRT in CH roots were more expressed either in stalks or in hydroponics, suggesting that this expression profile is under genotype control, independent of the colonization with diazotrophic bacteria. Besides the NRT, one DET annotated as AMT1;2 that encodes an ammonium transporter was also more expressed in the CH stalk shoot.

Nitrate reductase (NiR) is the key regulatory enzyme of the nitrate-assimilation pathway, by reducing nitrogen absorbed as nitrate (NO_3_^−^) to nitrite (NO_2_^−^), which is reduced to ammonium (NH_4_^+^), leading to plant N assimilation [51,52]. In addition to NRT, two NiR transcripts, the NIA1 and NIA2, also seem to be more active in CH germinated stalks, with one DET in the root and one DET in the shoot, respectively.

As members of the NPF family can transport diverse components as substrates besides nitrate, they could participate in diverse biological processes including plant growth, development, and adaptation to environmental changes [53]. Several NPF family members that are more expressed in CH tissues were described to be involved in stress responses in other plant species, such as NPF5.1–3, NPF4.5, NPF4.6, NPF7.3, NPF 8.2 and NRT3.2. AtNPF5.2 is needed for defense against some virulent bacterial pathogens in Arabidopsis [54]. NPF4.5, NPF4.6, and NPF8.2 could also be involved in stress responses, because they are capable of transporting ABA in addition to NO_3_^−^ [49,55], which is a stress hormone that accumulates under different abiotic and biotic stresses [56]. In addition, the Atnpf4.6 mutant showed a reduction in ^13^N export when soil N availability was low; however, when the N availability was adequate, the ^13^N export in the mutants was similar to the wild type. This suggests that AtNPF4.6 might be induced in low N conditions and support a function in stress tolerance, as well as low N tolerance [57]. A role for NPF7.3 in stress tolerance has also been proposed, and downregulation enhanced the tolerance to various stresses [58]. In *A. thaliana*, NRT3.2 is involved in stomatal regulation, since it is expressed in guard cells when in biotic stress [59]; however, the function remains unclear. As beneficial and pathogenic bacteria share several features of recognition, and NPF family members that were more expressed in CH were already described to be induced in a pathogen infection, it could suggest that this process must not be fine-tuned for a proper response in the CH genotype.

Higher mRNA levels of NRT1.1 and ScNIA2 in stalk shoots of CH compared with SP were confirmed by qRT-PCR, validating the RNA-seq data (Figure 4). The data suggest that N assimilation as nitrate, through nitrate transporters (NRTs), could be more active in the CH of germinated stalks.

#### 2.4.2. Amide Amino Acid Metabolic Pathway

Plant assimilation of fixed N can occur via the amide amino acid metabolic pathway that converts ammonium into nitrogen-transporting amino acids by the glutamine synthetase (GS)/glutamate synthase (GOGAT) cycle, or by the enzyme glutamate dehydrogenase (GDH) [60]. Most of the DETs that were annotated as components of the ammonium-assimilation pathway were more expressed in SP than in CH, such as almost all transcripts homologous to GS (with three DETs in the root and seven DET in the shoot) and to GOGAT (with two DETs in the root and one DET in the shoot) (Figure 6a,b and Table S6). The enzyme asparagine synthetase (AS) that catalyzes the synthesis of asparagine, an important nitrogen-transporting amino acid in several plant species, was also more expressed in SP (with eight DETs in the shoots and six DETs in the roots). The data suggest that ammonium assimilation in germinated stalks is more activated in the highly efficient BNF genotype, both in roots and shoots (Figure 6a,b and Table S6).

Higher expression levels of ScGS1a, ScGS1b, ScGS1c and GLT1 in stalk roots and shoots of SP compared with CH were confirmed by qRT-PCR, validating the RNA-seq data (Figure 4). In addition, biochemical analyses of enzyme activities were carried out to functionally validate the transcriptome data. Since GS enzyme activities can be modulated post-transcriptionally, they were measured in the leaves of germinated stalks. As observed in Figure 6c, GS activities were higher in the more efficient BNF genotype than in CH (3.89 ± 0.59 in SP and 3.19 ± 0.54 in CH), functionally validating the expression analysis data. Bacterial nitrogenase activities were also quantified in the same plant materials and compared between SP and CH. As observed in Figure 6c, bacterial nitrogenase activities were also higher in the more efficient BNF genotype than in CH (4.30 ± 1.81 in SP and 1.49 ± 1.33 in CH), corroborating the GS activities observed in the contrasting genotypes. Previous studies on differential EST expression of the sugarcane SP genotype, free of microorganisms and in vitro inoculated with endophytic diazotrophic bacteria, have indicated that N metabolism was activated in SP plants inoculated with the diazotrophic bacteria *G. diazotrophicus* and *H. seropedicae*. mRNA levels of a cytosolic GS (scGS1.b) were higher in SP than in CH, in mature leaves from field-grown plants [20]. In addition, in the IAC95-5000 sugarcane genotype, which is considered less efficient for diazotrophic bacterial inoculation, similarly to CH, the activity of glutamine synthetase was unaffected by PGPB [61]. The enzyme AS, which catalyzes the synthesis of the nitrogen-transporting asparagine, was also more expressed in SP, further suggesting that N assimilation was more active in SP than in CH. Similarly, the RB86-7515 sugarcane genotype, which responds better to inoculants with diazotrophic bacteria, showed an increase in glutamine and asparagine when inoculated by a mixed inoculum containing the *G. diazotrophicus* strain PAL5 and *H. seropedicae* [62].

#### 2.4.3. Amide Amino Acid Metabolic Pathway

The importance of nitrogen metabolism is highly related to the adaptation of plants to stress [63]. To grow in adverse conditions, plants have to adapt their physiological processes, in particular nitrogen metabolism; this requires major changes, including changes in the associated metabolic networks with amino acids [63,64,65]. More remarkably, the amino acid composition is modified when the plant is in a stress condition and is characterized by a high accumulation of specific amino acids involved in plant stress tolerance [65,66]. Thus, in addition to the important genes involved in nitrogen transport and assimilation, 468 and 275 DETs annotated as genes involved in amino acid metabolism were found in sugarcane shoots and roots, respectively (Figure 6 and Table S6). They included DETs annotated as alanine aminotransferase (five in the shoot), alcohol dehydrogenase (three in the root and six in the shoot), aspartate aminotransferase (three in the root and six in the shoot), aspartate kinase (two in the root and four in the shoot), glutathione peroxidase (four in the root and six in the shoot), glutathione reductase (two in the shoot), glutathione S-transferase (28 in the root and 73 in the shoot), among many others (235 in the root and 366 in the shoot). Most of them were more expressed in SP (173 in the root and 329 in the shoot). This could suggest that the amino acid pathway was more activated in the more responsive BNF genotype. In addition, several studies have already been carried out to investigate many of these genes, showing an important involvement with plant growth, yield and NUE increase, and stress tolerance [67,68,69,70,71,72,73,74,75]. Corroborating our data, the inoculation with beneficial bacteria enhanced the amino acid metabolism and increased glutamine, asparagine, glutamic acid, serine, proline, threonine, aspartic acid, and other amino acids in sugarcane [62,76]. In addition, sugarcane plants inoculated with the PGPB *Burkholderia anthina* MYSP113 also exhibited regulated amino acid pathways, showing several upregulated sequences [77].

### 2.5. Auxin Hormonal Pathway

Plant hormones are key signaling molecules that regulate plant growth and response to microbial interactions [78]. It has already been observed that some of the diazotrophic bacteria can produce phytohormones, such as IAA [79,80]. In this work, differential expression analyses between the contrasting genotypes identified hormone metabolism among the most represented functional categories (Figure 2c,d). Thus, the differential regulation of the auxin phytohormone pathway in the BNF-contrasting genotypes was investigated, by searching for DETs from biosynthesis, signaling and response pathways.

**Figure 7:**
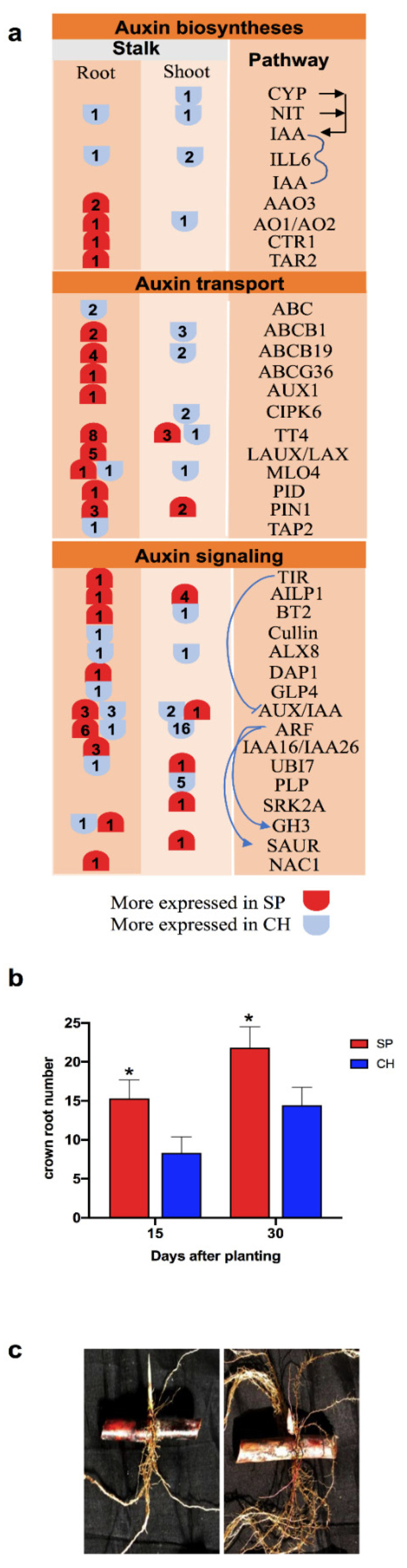
Evaluation of differential auxin pathway regulation between BNF-contrasting genotypes. (**a**) Schematic representation of auxin pathway showing the DET identified related to auxin biosynthesis, auxin transporter and auxin signaling in roots and shoots of germinated stalks. In red are represented the transcripts more expressed in SP and in blue are represented the ones more expressed in CH. (**b**) Evaluation of crown roots in the stalk plants (naturally colonized by endophytes). (**c**) Representative photos of germinated stalks 15 days after planting of CH (left) and SP (right) demonstrate the differences observed in the quantitative analysis. Bars represent mean ± standard deviation of three biological replicates. Asterisks (*) represent significant difference between SP and CH at the same time point through t-test (*p* < 0.05).

Different auxin biosynthetic routes in plants contribute to the pool of auxin in the plant tissues, such as Indole-3-acetaldoxime (IAOx), Indol-3-acetamide (IAM), Indol-3-Pyruvic acid (IPyA) and Indole-3-acetaldehyde (IAAld) pathways [81]. IPyA and IAAld pathways seem to be more active in SP than in CH roots, since key enzymes of these pathways—Tryptophan Aminotransferase Related 2 (TAR2, 1 DET) and Abscisic Aldehyde Oxidase 3 (AAO3, 2 DET)—were more expressed in SP than in CH (Figure 7a and Table S7). On the other hand, the IAOx pathway seems to be more active in CH, as one transcript annotated as a Nitrilase 4 (NIT4) was more expressed in this genotype (Figure 7a and Table S7). Final auxin levels also depend on the balance of the formation of IAA-amino acid conjugates, which are generally considered inactive, and the activity of hydrolases that convert the conjugates to free IAA [81]. mRNA levels of either an enzyme involved in the formation of auxin–amino acid conjugates—GH3; T1 (one DET)—and the IAA-amino acid hydrolases—ILL6 (one DET)—were higher in the roots of CH than in SP; in contrast, one transcript annotated as a GH3—T2—was more expressed in the SP roots. Most auxin transporters showed higher levels of expression in SP than in CH, as observed for Chalcone and stilbene synthase (TT4, eight DETs), Pin-formed 1 (PIN1, three DETs), ATP-Binding Cassette B19 (ABCB19, four DETs), ATP-Binding Cassette B1 (ABCB1, two DETs), Auxin Resistant 1 (AUX1, one DET), Seven transmembrane MLO family (MLO4, one DET), Like Auxin Resistant 1 (LAX1, three DETs), like AUX1 3 (LAUX3, one DET), Protein kinase superfamily (PID, one DET) and Like Auxin Resistant 2 (LAX2, one DET), except for the ABC transporter family (ABC, two DETs), Transporter associated with antigen processing 2 (TAP2, one DET), MLO4 (one DET) and ABC-2 and Plant PDR ABC-type transporter family (ABCG36, one DET), which exhibited higher mRNA levels in CH (Figure 7a and Table S7).

Auxin signaling and response depends on its recognition by the intracellular receptor of the Auxin Signaling F-BOX/Transport Inhibitor Response (AFB/TIR) family [82]. Through this interaction, AUX/IAA (Indole-3-acetic acid inducible), a repressor of the pathway, is led to degradation by ubiquitination, which allows the transcriptional activation of auxin-responsive genes by ARFs (Auxin Response Factor) [82]. All of the DETs positively involved in auxin signaling, such as TIR1 (1 DET), ARF2 (1 DET) and ARF3 (1 DET), showed higher levels of expression in SP than in CH (Figure 7a and Table S7). However, two transcriptional repressors, IAA16 (2 DET) and IAA26 (1 DET), were also more expressed in SP roots. Possibly, the higher levels of GH3, IAA16 and IAA26 in SP roots could be related to their function as modulators of auxin signaling, triggering negative-feedback controls for proper auxin homeostasis [83,84]. Finally, the transcriptional factor NAC1, which is induced by auxin and is involved in auxin signaling to promote lateral root development in *A. thaliana* [85] and in maize [86], was also more expressed in SP stalk roots than in CH.

The transcriptome data suggest that in the roots of the high-BNF genotype, which was highly colonized with endophytic diazotrophic bacteria, auxin biosynthesis, transport and signaling transduction were more active than in the roots of the low-BNF plants. Auxin regulates almost every aspect of plant growth and development [87,88]. In roots, the most well characterized auxin-associated phenotypes are the increase in root hair length, the effect of auxin on primary root growth, the increase in the number of lateral root primordia, and the response to gravity [89,90,91]. As shown in Figure 7a, members of auxin signaling known to be positive regulators of root development, such as TAR2, PIN1, AUX1 and TIR1, were more expressed in SP. In addition, key regulators of root architecture (ARF2, ARF3, NAC1) showed expression profiles that were compatible with the greatest root development observed in SP, which is the most efficient BNF genotype. Differential expression levels of TAR2, AUX1, ABCB19, TIR1, ARF2 and NAC1 in the stalk roots of SP compared with CH were confirmed by qRT-PCR, validating the RNA-seq data (Figure 4).

In Arabidopsis, the overexpression of TAR2 increases LR numbers under both high and low nitrogen conditions [92]. Rice PIN1 plays an important role in auxin-dependent adventitious root emergence and tillering [93]. Disruption in auxin transport by AUX1 mutation results in altered IAA distribution and consequently the inhibition of LR formation in Arabidopsis [94]. Auxin receptor mutants, including TIR1, have altered LR phenotypes [95]. Downstream of TIR1, NAC1 acts in lateral root formation [85]. NAC1 sense or antisense transgenic lines show an increase or reduction in lateral roots, respectively. In addition, TIR1-induced lateral root development is blocked in the antisense NAC1 transgenic line, and NAC1 overexpression can restore lateral root formation in the tir1 insertion mutant. The overexpression of the maize NAC1 ortholog, ZmNAC1, in the heterolog system of Arabidopsis resulted in an increase in the number of lateral roots in comparison to wild type plants [86]. However, a functional analysis of the transcription factors ARF2 and ARF3 in Arabidopsis, whose putative homologs were more expressed in SP, demonstrated the inhibitory effect of these proteins in LR development [96]. Our data did not allow us to assign the sugarcane genes as functional orthologs, and further analysis might be performed. In addition, SP showed higher levels of expression of some members of the AUX/IAA family, which were characterized as repressors of the auxin effect on root development of other plant species, and they could be involved in the fine regulation of the pathway.

One of the well-known beneficial outcomes of the plant–endophytic diazotrophic bacteria association is root growth promotion [21,97,98]. To biologically validate the auxin gene expression profile observed in the transcriptome data, root development during stalk germination was analyzed in the contrasting BNF genotypes. As shown in Figure 7c, in germinated stalks naturally colonized with diazotrophic bacteria, the root system in SP was more developed than in CH. In addition, the SP genotype presented 84% and 51% more crown roots than CH 15 and 30 days after germination, respectively (Figure 7b).

**Figure 8:**
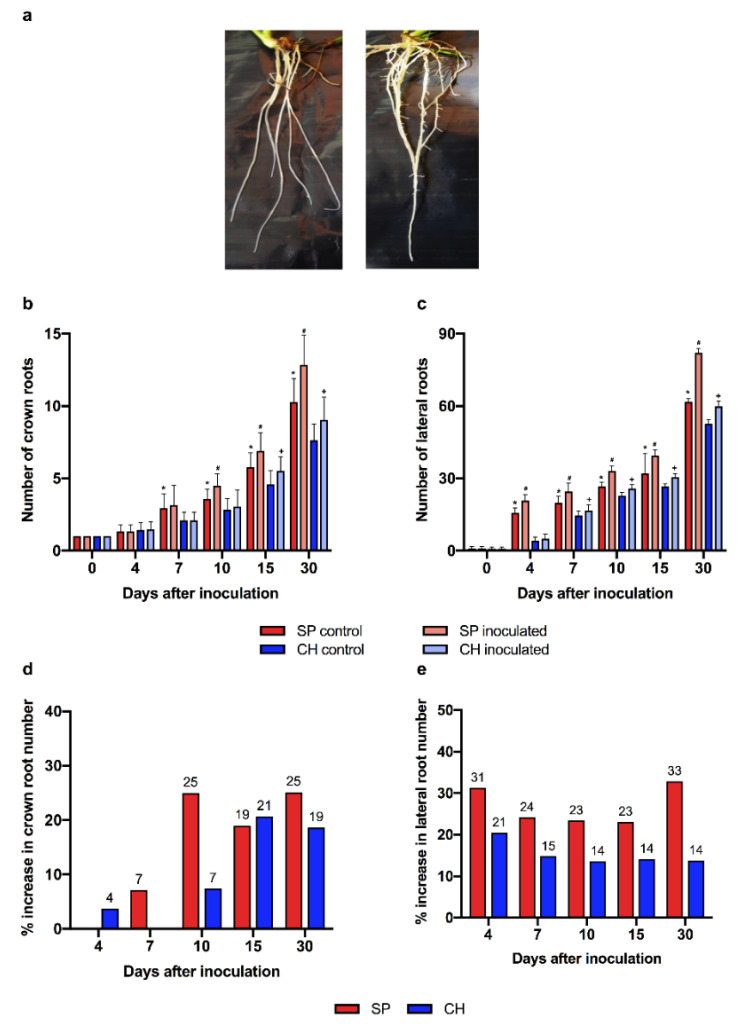
Analysis of roots between the contrasting genotypes grown in hydroponics. (**a**) Representative photos of diazotrophic-free plants grown four days after transfer to hydroponics demonstrate the differences observed in the quantitative analysis of control plants. Evaluation of crown (**b**) and (**c**) lateral roots, and % of increase in (**d**) crown and lateral root number (**e**) in plants inoculated with *G. diazotrophicus*. Analyses were performed 0, 4, 7, 10, 15 and 30 days after inoculation. The symbol (*) indicates significant differences between SP and CH control plants at the same time point. The symbol (#) indicates significant differences between control and inoculated plants of the same genotype at the same time point. The (+) sign indicates significant differences between SP and CH inoculated plants at the same collection point. Bars indicate mean ± standard error of three independent experiments.

To investigate root growth modulation due to intrinsic genotype variations, in vitro micro propagated plantlets free of microorganisms were transferred to hydroponics where their root development was analyzed. As shown in Figure 8a, the SP root system was more developed than in CH. Four and seven days after growing in hydroponics, the SP plants already presented a greater number of crown (Figure 8b) and lateral roots (Figure 8c), respectively, compared to CH plants. The quantitative evaluation of root development demonstrated that at these time points SP had 39% more crown roots (Figure 8b) and 284% more lateral roots than CH (Figure 8c). Up to thirty days after growing in hydroponics, this difference was still significant. While SP plants had an average of 10 crown roots and 62 lateral roots, CH showed approximately 8 and 53, respectively (Figure 8b,c). The data showed that in plants free of diazotrophic bacteria, the root system in SP was more developed than in CH, supporting that genotype-specific genetic factors are involved in the differences in root development and architecture observed between the BNF-contrasting genotypes.

Next, we analyzed the role of the diazotrophic bacteria association in the root development of the two genotypes. In vitro micro propagated plants free of microorganisms were transferred to cultivation in hydroponics, and then inoculated with *G. diazotrophicus*. Time-course analyses of the root development showed root growth promotion after inoculation with diazotrophic bacteria (Figure 8b,c). The CH genotype showed a delay in the promotion of the growth of crown (Figure 8b) and lateral roots (Figure 8c) compared to SP in all of the experimental points analyzed. Only 15 and 10 days after inoculation were significant increases in the number of crown (21%) and lateral (21%) roots, respectively, observed in the CH plants. The maximum promotion of crown and lateral roots in SP occurred 30 days after inoculation (25% and 33% respectively). In the inoculation of the CH genotype, maximum levels of growth promotion of crown (21%) and lateral (21%) roots were observed 15 and 4 days after inoculation, respectively (Figure 8d,e). As shown in Figure 8d,e, the promotion of sugarcane root growth by the diazotrophic bacteria was more pronounced in SP (efficient association) than in CH (inefficient association).

**Figure 9:**
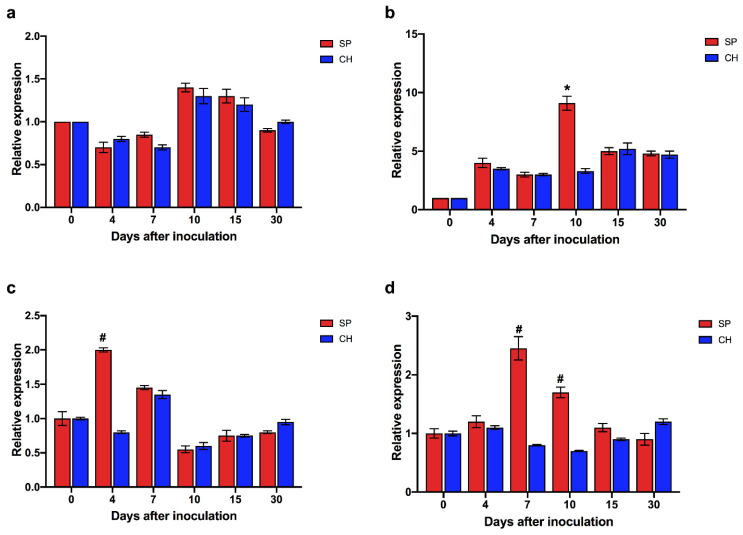
Expression of the ScTIR1 and ScNAC1 genes in roots of plants of the SP and CH genotypes not inoculated and inoculated with diazotrophic bacteria. (**a**,**c**) represent the relative expression by qRT-PCR of the (**a**) ScTIR1 and (**b**) ScNAC1 genes in roots of plants free from diazotrophic bacteria of the BNF-contrasting genotypes. (**c**,**d**) represent the relative expression by qRT-PCR of the (**c**) ScTIR1 and (**d**) ScNAC1 genes in roots of control and *G. diazotrophicus*-inoculated plants of the BNF-contrasting genotypes. Relative mRNA levels were analyzed by qRT-PCR and were normalized by the levels of the 28S and GAPDH normalizers and (**a**) the 0-day point or (**b**) the uninoculated control on the same day of collection was used as a reference for the calculation of relative expression. Each root RNA sample was prepared from a pool of 15 plants, and the figure shows the average of three biological replicates where technical triplicates of the qRT-PCR reactions were performed. Statistical analyses were performed by t-test with *p* < 0.05. The symbol (*) indicates significant differences in the expression of ScNAC1 in roots of plants free of diazotrophic bacteria between SP and CH at the same time point. The (#) sign indicates significant differences in expression between control and inoculated plants of the same genotype and at the same collection point. Bars indicate mean ± standard deviation of three independent experiments.

To better understand the dynamics of the expression of auxin pathway genes during root development and during the association with diazotrophic bacteria, qRT-PCR was performed to assess the expression of ScTIR1 and ScNAC1 genes (Figure 9). During development, there was no significant difference in ScTIR1 expression between plants of the SP and CH genotypes that were free of diazotrophic bacteria (Figure 9a). Both genotypes showed the maximum point of expression 10 days after transfer to hydroponics. In plants associated with *G. diazotrophicus*, the ScTIR1 expression peaked four days after inoculation in SP, and it was not regulated in CH (Figure 9c). A difference in ScNAC1 expression was observed between the SP and CH genotypes free of diazotrophic bacteria 10 days after transfer to hydroponics (Figure 9b), when ScNAC1 expression peaked in the SP plants. Inoculation significantly increased ScNAC1 expression after seven days in SP, afterwards the mRNA levels reduced. The CH genotype did not show significant regulation of ScNAC1 expression after inoculation with *G. diazotrophicus* (Figure 9d). The expression profiles of ScTIR1 and ScNAC1 suggest that diazotrophic bacteria inoculation anticipated auxin responses in SP roots.

**Figure 10:**
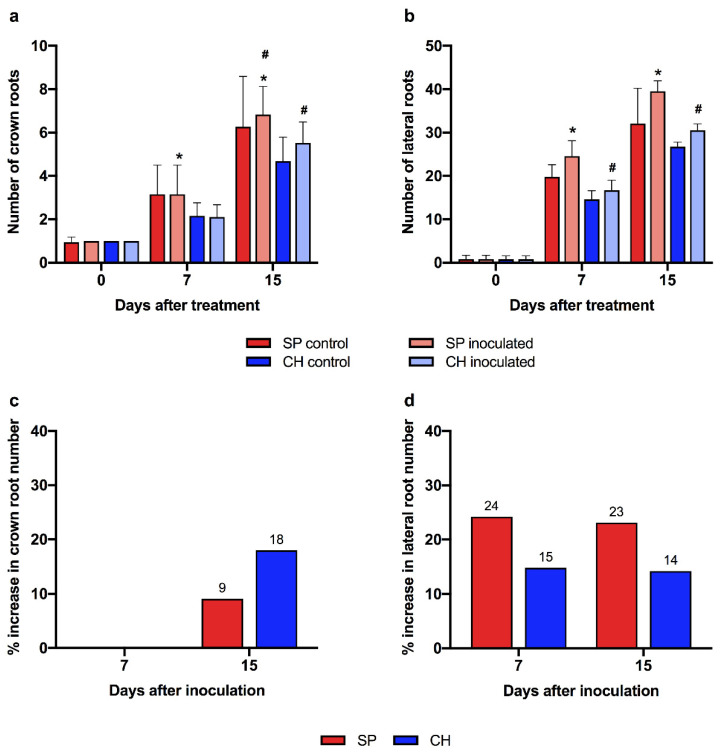
Analysis of root growth in the contrasting genotypes treated with 2 mM NAA in hydroponics. Evaluation of (**a**) crown and (**b**) lateral roots, and % of increase in (**c**) crown and (**d**) lateral root number in plants treated with 2 mM NAA. Analyses were performed 0, 7 and 15 days after treatment. The symbol (*) indicates significant differences between SP and CH control plants at the same time point. The symbol (#) indicates significant differences between control and NAA treated plants of the same genotype at the same time point. Bars indicate mean ± standard error of three independent experiments.

Finally, the responsiveness of root development to auxin was evaluated in the BNF-contrasting genotypes. Time-course analyses of root development showed root growth promotion after 1-naphthaleneacetic acid (NAA) treatment (Figure 10a–d). The maximum promotion of crown and lateral roots in SP occurred 15 and 7 days after inoculation (9% and 24% respectively). In the NAA treatment of the CH genotype, it showed maximum levels of growth promotion of crown (18%) and lateral (15%) roots 15 and 7 days after treatment, respectively (Figure 10a–d). As shown in Figure 8d,e, the promotion of sugarcane root growth by the diazotrophic bacteria was more pronounced in SP (efficient association) than in CH (inefficient association). Both genotypes showed root growth promotion by NAA treatment. However, the rate of increase was higher in SP plants than in CH (Figure 10d), suggesting that this genotype is more responsive to auxin, corroborating the gene expression and phenotypic analysis data.

Remarkably, the dynamics of expression of auxin signaling genes in the BNF-contrasting genotypes positively correlated with the timing of their root development, both in inoculated and non-inoculated plants, as the CH genotype showed a delay in the growth of crown and lateral roots compared to SP in both conditions. Thus, the regulation of the expression of auxin pathway genes in SP, such as ScTIR1 and ScNAC1, during association with diazotrophic bacteria may represent an important molecular mechanism for growth promotion modulated by the bacterial association. In addition to being involved in lateral root development, transcription factors that are members of the NAC gene family have been suggested to be involved in the regulation of the transcriptional reprogramming associated with plant stress responses [99]. The overexpression of ZmSNAC1 in *A. thaliana* enhanced tolerance to drought and low-temperature stress when compared to the control plants [100]. In transgenic rice, the overexpression of Os03g60080/SNAC1 increased grain yield (up to 34%) under drought stress [101].

Altogether, the data suggest that the contrasting root development and auxin response pathway profiles between SP and CH might result from two levels of regulation: intrinsic genotypic variations between SP and CH, plus the efficiency of the association with diazotrophic bacteria.

## 3. Materials and Methods

### 3.1. Plant Material for Library Construction and RNA Extraction

Stalks of SP70-1143 and Chunee genotypes, kindly provided by Embrapa Agrobiologia (Seropédica, RJ), were germinated in a mixture of sand and vermiculite in 2:1 ratio, respectively. Watering was performed twice per week and the temperature was maintained between 25 °C and 30 °C with an irradiance of 60 μmol photons m^−2^ s^−1^ for 12 h d^−1^. Root and shoot tissue samples were separately collected 15 days after planting.

A hydroponic system was established to generate diazotrophic-bacteria-free plant material. SP70-1143 and Chunee plantlets obtained from sterile in vitro culture of meristems (provided by Centro de Tecnologia Canavieira—CTC) were transferred to Hoagland’s solution [102] and the material was collected 30 days after transferring to hydroponic culture. Root and shoot tissues were collected separately, and before freezing, samples were washed in water/alcohol/water (1 min for each).

All 16 collected samples were frozen in liquid nitrogen and stored in a freezer at −80 °C until extraction. For RNA-seq library construction, samples collected from fifteen plantlets were pooled and total RNA from two pooled samples were extracted from frozen material according to [103]. Quality and quantification of RNA samples was performed using Agilent 2100 Bioanalyzer TM (Agilent Technologies, Aplo Alto, CA) and verified by agarose gel electrophoresis. Total RNA of two biological replicates of each plant material were used for deep sequencing at Fasteris Life Sciences SA (Plan-les-Ouates, Switzerland). Abbreviations of all libraries sequenced are summarized in Table S1.

Evaluation of colonization by diazotrophic bacteria in all samples was assessed by qRT-PCR.

### 3.2. Plant Material for Experiments in Hydroponic Culture and Root Development Analysis

The hydroponic system was also used to perform hormone treatment and inoculation experiments. SP and CH plantlets obtained from sterile in vitro meristem culture were transferred to Hoagland’s solution and submitted to different treatments. For inoculation experiments, sugarcane plantlets were inoculated with a suspension of *G. diazotrophicus* strain PAL5. The control and inoculated plants had their root system analyzed by quantification of crown and lateral roots 0, 4, 7, 10, 15 and 30 days after inoculation (DAI). At the same time points, roots from 15 plants were collected in three pooled samples and frozen in liquid nitrogen for further RNA extraction. For hormone treatment experiments, NAA was added to Hoagland’s solution to a final concentration of 2 mM. The control and NAA-treated plants had their root system analyzed by quantification of crown and lateral roots 0, 7, and 15 days after treatment (DAT).

Root architecture of germinated stalks was evaluated. SP and CH stalks were germinated in a mixture of sand and vermiculite in a 2:1 ratio and submitted to quantification of their number of crown roots 15 and 30 days after planting.

### 3.3. Bacteria Inoculation and Evaluation of Plant Colonization

To prepare bacterial inoculation suspension, *G. diazotrophicus* strain PAL5 was grown on a Petri dish with LGI-P solid medium (sucrose or sugar 100 g/L, K_2_HPO_4_ 0.2 g/L, KH_2_PO_4_ 0.6 g/L, MgSO_4_.2H_2_O 0.2 g/L, CaCl_2_.2H_2_O 0.02 g/L, Na_2_Mo_4_.2H_2_O 0.002 g/L, Bromothymol blue 5 mL/L (0.5% solution in KOH 0.2 N), FeCl_3_.6H_2_O 0.01 g/L, pH 5.5–6.0). Bacterial suspensions from one colony were grown in 5 mL liquid DYGS (glucose 2 g/L, peptone 1.5 g/L, yeast extract 2 g/L, K_2_HPO_4_ 0.5 g/L, MgSO_4_.7H_2_O 0.5 g/L and glutamic acid 1.5 g/L, pH 6.0) for two days at 28–30 °C with agitation (120 rpm) as a pre-inoculum. A volume of 1 mL from pre-inoculum was added to each 100 mL liquid DYGS and allowed to grow overnight under agitation at 28–30 °C to form the inoculation solution. Bacterial suspensions with OD_600nm_ equal to one were added to 1x Hoagland’s solution at a proportion of 1:30. The same amount of medium without bacteria was added in Hoagland’s solution as mock controls. *G. diazotrophicus* PAL5 was kindly provided by EMBRAPA, Seropédica, RJ.

To evaluate bacterial colonization, whole plants were washed with sterile water for one minute, with sterile 70% ethanol for one minute and with sterile water for one minute. After that, plants were macerated in sterile saline solution (100 g/L, K_2_HPO_4_ 0.2 g/L, KH_2_PO_4_ 0.6 g/L, MgSO_4_.2H_2_O 0.2 g/L, CaCl_2_.2H_2_O 0.02 g/L, Na_2_Mo_4_.2H_2_O 0.002 g/L) in the proportion 9 mL of saline solution for 1 g of plant. Homogenate was used for serial dilution from 10^−1^ to 10^−9^. A sample of 100 uL of all dilutions was inoculated in 5 mL of LGI-P semi-solid medium (sucrose or sugar 100 g/L, K_2_HPO_4_ 0.2 g/L, KH_2_PO_4_ 0.6 g/L, MgSO_4_.2H_2_O 0.2 g/L, CaCl_2_.2H_2_O 0.02 g/L, Na_2_Mo_4_.2H_2_O 0.002 g/L, Bromothymol blue 5 mL/L (0.5% solution in KOH 0.2 N), FeCl_3_.6H_2_O 0.01 g/L, pH 5.5–6.0). Bacterial growth was evaluated every day until growth pellicle visualization.

### 3.4. Library Construction, Sequencing and De Novo Assembly

For construction of mRNA libraries, first-strand cDNA was generated using random hexamer-primed reverse transcription. After second strand cDNA synthesis and adaptor ligation, cDNA fragments of approximately 200 bp were isolated by gel electrophoresis. cDNA fragments were amplified by 15 cycles of PCR and deep sequenced on the Illumina Genome Analyzer (GA-IIx) system using a single-end 100 cycle protocol, following the manufacturer’s protocol available at http://www.fasteris.com, accessed on 10 July 2012. Demultiplexing was used prior to generation of fastq sequence files by separating the libraries according to their indexes. Raw reads in the fastq format were cleaned using quality trimming and quality filtering as implemented in the FASTX Toolkit (version 0.0.13, http://hannonlab.cshl.edu/fastx_toolkit/). For quality trimming, a quality threshold of 30 was used with a minimum read length of 20 nucleotides. For quality filtering, the minimum quality score was set to 20 in a minimum percent of bases of 90%.

After trimming low-quality sequences (Q30), Illumina sequencing data obtained from all samples were used for de novo assembly using software tool VELVET (version 1.0.12) [104] in combination with OASES (version 0.1.15) [105]. Different “k-mer” (35–49) were used to optimize the assembly process, searching for an increase in both contig contiguity and in transcript diversity, and after that hash size 47 was chosen. The average insert size was set to 200 bases with a standard deviation of 10%. A minimum size of 100 bases was set for the contigs and the coverage cut-off for contigs was set to 6X. Only contigs with more than 100 bp were maintained in the final dataset. The OASES tool of VELVET package was used to solve ambiguities that may have occurred in splicing sites. The contigs were organized into clusters called loci, and these were fragmented because of splice variations and partial assemblies.

Therefore, in the reference transcriptome constructed, all loci are detected with possible transcripts enumerated for each of them. Sequences generated were identified by the locus ID and transcript number correlated to it, e.g., Locus_1_Transcript_3/4_Confidence_0.429, where a locus indicates a suspected genomic locus, transcript 3/4 indicates that this is isoform 3 of 4 identified, and confidence indicates the fraction of reads within this locus which support this isoform. Only transcripts above 100 bp were considered.

The datasets generated for this study can be found in SRA/NCBI (https://www.ncbi.nlm.nih.gov/bioproject/PRJNA226750).

### 3.5. Read Mapping and Differential Expression Analysis

For expression analysis, reads of each library were mapped against the reference transcriptome and counted at the transcript level using MAQ program [106]. The following parameters were considered: total alignment of reads with up to 2 mismatches in the first 24 bases of the assembled transcript and extension to other bases.

To select differentially expressed transcripts, read counts were normalized as RPKM (read per million fragments per kilobase), by multiplying the total number of reads by 1 billion (1-million-time 1000) and dividing by the annotation size (a total read number multiplying by the transcript length) [107]. This allows comparison between the samples. A Fisher’s Exact test with a *p*-value cut-off < 0.05 was performed on every combination of the 16 libraries with Bonferroni correction. We used a Log2 Fold change (Log2FC) to create a transcriptomic dataset with comparisons of interest. The fold change is calculated dividing the RPKMs from a condition of interest by the control. This way we measure the difference of expression between SP and CH samples.

For exclusively expressed sequence evaluation, transcripts that were covered by at least 1 read in 5 CH samples, and never covered by any SP samples that were considered as exclusively expressed in Chunee and vice versa.

### 3.6. Functional Annotation of Sugarcane Transcripts

The sugarcane transcriptome sequences were evaluated against proteins of Viridiplantae UNIPROT database (https://www.uniprot.org/uniprot/?query=viridiplantae&sort=score, accessed on 20 November 2020) using the BLASTX and an e-value cut-off threshold of ≤ 1 × 10^−5^. The Viridiplantae transcripts of RT1 were mapped against sugarcane genome sequences available in public databases, using the BLASTN tool with an e-value cut-off threshold of ≤ 1 × 10^−5^. Likewise, conservation of RT1 sequences was analyzed with proteome databases of Arabidopsis, rice, maize and sorghum using the BLASTX tool.

Sugarcane cDNA sequences were retrieved from the Sugarcane genomes Index (https://sugarcane-genome.cirad.fr, accessed on 15 July 2018; and https://bioinformatics.psb.ugent.be/plaza/versions/plaza_v4_5_monocots, accessed on 10 October 2018). The *Sorghum bicolor* (Sbicolor_454_v3.1.1, 1 February 2017), *Oryza sativa* (Osativa_323_v7.0 27 November 2015), *Zea mays* (Zmays_493_RefGen_V4, 21 October 2017) and *Arabidopsis thaliana* (Athaliana_167_TAIR10, 12 January 2014) protein data sets were obtained from Phytozome (http://www.phytozome.net, accessed on 10 October 2020).

All the transcripts were subjected to Gene Ontology and Kyoto Encyclopedia of Genes and Genomes analysis. For the enrichment analysis of functional categories, DEGs were classified according to Gene Ontology terms and KEGG pathway using OmicShare tools (http://www.omicshare.com/tools, accessed on 15 November 2020). The significance levels of the GO terms in the three functional categories—biological process, molecular function, and cellular component. A GO term or KEGG pathway was considered significantly enriched if the *p*-value < 0.05.

Genes encoding proteins involved in auxin biosynthesis, receptor, signaling, and transporter were identified using the Arabidopsis Hormone Database v.2 [108]. Genes encoding proteins involved in nitrogen metabolism and amino acid metabolism were identified using KEGG annotation. Genes related to plant receptor (microorganism recognition) were identified using MAPMAN [109].

### 3.7. RNA Expression by qRT-PCR

Total RNA of three biological replicates of each plant material were used for qRT-PCR. Total RNA isolated from roots and shoots were treated with DNAse I (Biolabs). Reverse transcription was made using Taqman First Strand cDNA Synthesis Kit (Applied) using random hexamers as primers, following manufacturer’s instructions. To analyze gene expression profile, qRT-PCR reactions were performed with SYBR Green PCR Master Mix (Applied Biosystems). To each well, 2.5 μL of 3x diluted first strand cDNA, 5 μL of SYBR Green solution, 1 μL of the forward primer (10 μM) and 1 μL of reverse primer (10 μM) were added, along with 0.5 μL of sterile, ultrapure water to bring the final volume to 10 μL in each well. qRT-PCR reactions were performed using Applied Biosystems 7500 Real-Time PCR Systems, under standard conditions. The constitutive plant genes 28S ribosomal RNA (28S rRNA) and GAPDH were used as internal control: 5′-GCGAAGCCAGAGGAAACT-3′ (28S rRNA forward), 5′-GACGAACGATTTGCACGTC-3′ (28S rRNA reverse), 5′-CACGGCCACTGGAAGCA-3′ (GAPDH forward) and 5′-TCCTCAGGGTTCCTGATGCC-3′ (GAPDH reverse). To validate the expression pattern of DET identified from RNA-seq analysis, 29 specific primers were designed with Primer Express software (Table S8). The sequence for primer design for each selected transcript was carefully selected in order to be specific to each putative transcript. The results of qRT-PCR were analyzed using ΔCt quantitative method according to [110]. Statistical analyses were performed using t-test.

### 3.8. Glutamine Synthetase and Nitrogenase Activity

Enzyme activity of GS and nitrogenase was evaluated in 15-day germinated stalks. The GS activity was measured as described by [111]. Briefly, samples of fresh leaves were collected from 5 plants, from each genotype, macerated with liquid nitrogen to produce a fine powder to which 1.5 mL of extraction buffer was added. The mixture was centrifuged at 14,000 rpm in a refrigerated centrifuge at −4 °C for 30 min. The total protein content was determined by absorbance measurement using a spectrophotometer at 540 nm and with bovine serum albumin (Sigma) as a standard. Protein extracts were used to quantify the GS activity (GSa). The absorbance was measured at 540 nm using an Anthos Zenyth 200 ST microplate reader (Biochrom) with standard γ-glutamyl monohydroxamate (Sigma). GSa was expressed in μmol of γ-glutamyl monohydroxamate min^−1^ μg^−1^ protein. The nitrogenase activity was measured by the acetylene reduction assay as described by [112].

## 4. Conclusions

Several Brazilian sugarcane cultivars show high contributions of Biological Nitrogen Fixation, with the ability to grow with low nitrogen fertilizer inputs. The use of bio-inoculants formulated with endophytic and associative nitrogen-fixing bacteria appears to be a promising practice to improve this already successful association. However, the effectiveness of this natural fertilization is heavily dependent on soil conditions and plant genotype. The understanding of how genetic controls in different plant varieties are coordinated with the colonizing nitrogen-fixing bacteria can provide tools to improve the use of bio-inoculants in order to achieve higher levels of productivity.

In this work, differential transcriptome, physiological and biochemical approaches were used to unravel plant genes and pathways that are differentially regulated in sugarcane BNF-contrasting genotypes, during a natural association with diazotrophic bacteria. Two sugarcane genotypes were studied: the commercial cv. SP70-1143 with high rates of BNF and the wild non-commercial species *Saccharum barberi* (Chunee), with low rates of N fixed by BNF [3]. To simulate field conditions, where sugarcane is mainly propagated through plantlets germinated from stalks that become highly colonized by diazotrophic bacteria, root and shoot tissues of stalks, 15 days after germination, were used to construct RNA-seq libraries. A reference transcriptome of sugarcane of high quality was generated from Illumina RNA-seq libraries of two BNF-contrasting genotypes, both in the presence and absence of diazotrophic bacteria, which is available to the entire scientific community.

In addition, several differential transcriptomes were generated, which unveil important physiological features intrinsic to each genotype and those that could be related to the efficiency of the association with the diazotrophic bacteria. Physiological and biochemical analyses functionally supported the biological involvement of microorganism recognition (plant receptors), nitrogen metabolism and auxin signaling in the differences between the two sugarcane genotypes. Possibly, BNF-contrasting genotypes might offer a distinct environment for bacterial growth, and the diazotrophic bacteria could stay in a lower active physiological status when colonizing genotypes with lower BNF contributions. In addition, the diazotrophic bacterial colonization might differentially modulate nitrogen metabolism in each plant genotype, impacting how much it will benefit from the bacteria. Finally, the modulation of auxin response appears to be a key mechanism regulating the efficiency of the association in promoting plant development.

One of the major outputs of the studies on sugarcane association with diazotrophic bacteria is the development of tools to help to obtain novel plant varieties more responsive to N_2_-fixing bacteria. The molecular analysis of the association reveals sugarcane genetic controls involved in the first step of plant response to diazotrophic bacteria colonization [20,22,32,113]. Our results showed transcriptional differences between BNF-contrasting genotypes that may explain, at least in part, the differences in response to the association with diazotrophic bacteria that lead to diverse contributions of BNF for each plant genotype. A follow-up of these studies is important in order to increase knowledge on how the plant genotype controls the efficiency of the association in conditions closer to the natural environment of crop growth. Several of the genetic controls revealed for sugarcane might possibly be translated to other plant species. They could be used for the development of tools for optimization of plant growth and response to bio-inoculants, presenting a sustainable alternative to the use of chemical fertilizers, with positive economic and environmental impacts on agriculture.

## Figures and Tables

**Table 1:** Representation of sugarcane transcriptome in other plant databases. Sugarcane transcriptome *loci* were mapped against some plant species, and a high percentage of total proteins of these plants was represented.

Plant Species	Total Proteins ^a^	Mapped Proteins ^b^	Sugarcane *loci* ^c^	% Sugarcane *loci* ^d^
Arabidopsis	35,386	14,920	34,617	64.83
Rice	49,061	19,639	40,839	76.48
Maize	63,540	23,462	42,438	79.47
Sorghum	29,448	19,545	43,715	81.87

^a^ Total number of proteins annotated in public databases. ^b^ Number of proteins that map with sugarcane transcripts. ^c^ Number of sugarcane *loci* that map with proteins of other plant species. ^d^ Percentage of sugarcane *loci* that map with other plant species in relation to total number of sugarcane *loci.*

## Data Availability

The raw RNA-seq data are available in the National Center for Biotechnology Information (NCBI) Sequence Read Archive (https://www.ncbi.nlm.nih.gov/sra) under the BioProject accession number PRJNA226750 and BioSamples accession numbers SAMN02397351–SAMN02397366.

## Supplementary Materials

The following supporting information can be downloaded at: https://www.mdpi.com/article/10.3390/plants11151971/s1. Figure S1. KEGG pathway enrichment analysis. Genes were categorized according to the number of genes in each pathway in roots (a) and in shoots (b). The annotation within each category was performed using the OmicShare tool. Figure S2. Pattern of expression of DETs between SP and CH in hydroponics roots. Results are presented as the ratio of the pattern of expression of DET between SP and CH, showing log2 values in the transcriptome libraries and the qRT-PCR analyses. For the expression in the transcriptome libraries, results are presented as the ratio SP/CH of the RPKM of each transcript. Bars represent mean ± standard deviation of the relative mRNA expression in two biological replicates. For the qRT-PCR data, results are presented as the ratio of expression of each transcript (relative to GAPDH and 28S rRNA) in each sample. Bars represent mean ± standard deviation of the relative mRNA expression in three biological replicates (3 plants) and each biological replicate analyzed with three technical replicates. Figure S3. Exclusive transcripts identified in BNF contrasting genotypes. Patterns of expression of exclusive transcripts of SP or CH were evaluated by qRT-PCR in all 16 samples used in transcriptome sequencing analysis. The transcripts levels were normalized with plant rRNA and GAPDH in each sample. Each sample was representative of three plants, with three technical replicates and three biological replicates were evaluated. Table S1. Summary of the codes of all the libraries sequenced and representation of sequence results of the different libraries of sugarcane transcriptome. Table S2. Functional annotation of differentially expressed transcripts from sugarcane datasets and commonly expressed in both stalk and hydroponic datasets. In gray are represented the exclusive transcripts. Table S3. Gene ontology (GO) enrichment analysis of differentially expressed transcripts in roots and shoots. In gray are the GO terms represented in the Figure 2. Table S4. KEGG pathway enrichment analysis of DET, performed using Omicshare tools. Table S5. Annotated DET between SP and CH in germinated stalk tissues belonging to the Receptors function category. Table S6. Annotated DET between SP and CH germinated stalk tissues belonging to the N-metabolism and amino acid metabolism functional category. Table S7. Differentially expressed transcripts (DET) from sugarcane involved in plant hormone metabolism as identified by homology analysis with Arabidopsis thaliana hormone database (Jiang et al., 2010). Log2 fold changes of DET in stalk root and shoot and in root and shoot of plants cultivated in hydroponic, both from sugarcane BNF-contrasting genotypes. Table S8. Summary of specific primers designed for qRT-PCR analysis.
