# Supplementary material for: Sugarcane Genotypes with Contrasting Biological Nitrogen Fixation Efficiencies Differentially Modulate Nitrogen Metabolism, Auxin Signaling, and Microorganism Perception Pathways
Source: Plants (Basel). 2022 Jul 29;11(15):1971. doi: 10.3390/plants11151971 (PMC9370643; doi:10.3390/plants11151971)
Supplement: Supplementary file 1 [file plants-11-01971-s001.zip › plants-1810075-Figure S1-S3.pdf]

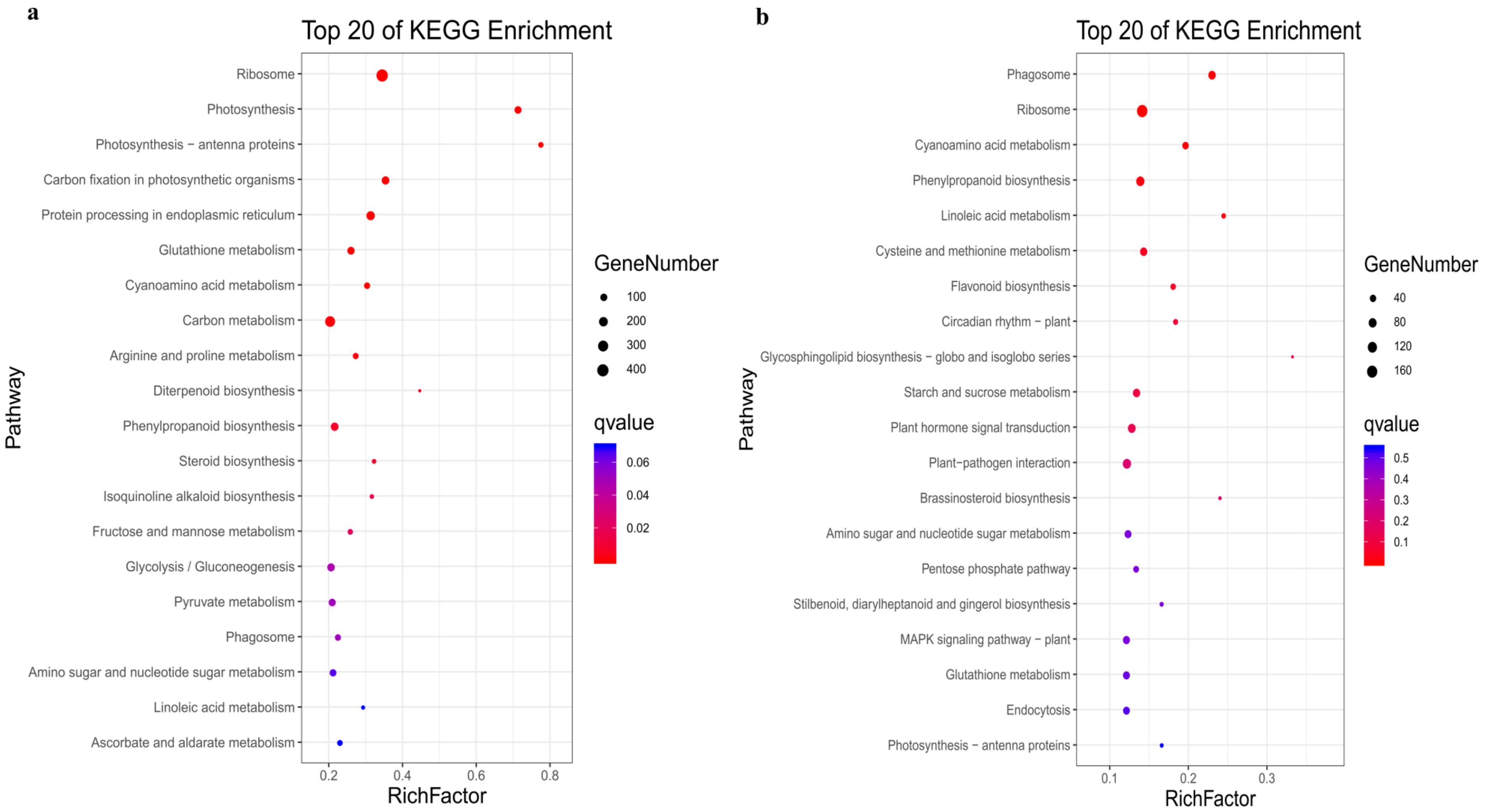

Figure S1. KEGG pathway enrichment analysis. Genes were categorized according to the number of genes in each pathway in roots (a) and in shoots (b). The annotation within each category was performed using the OmicShare tool.

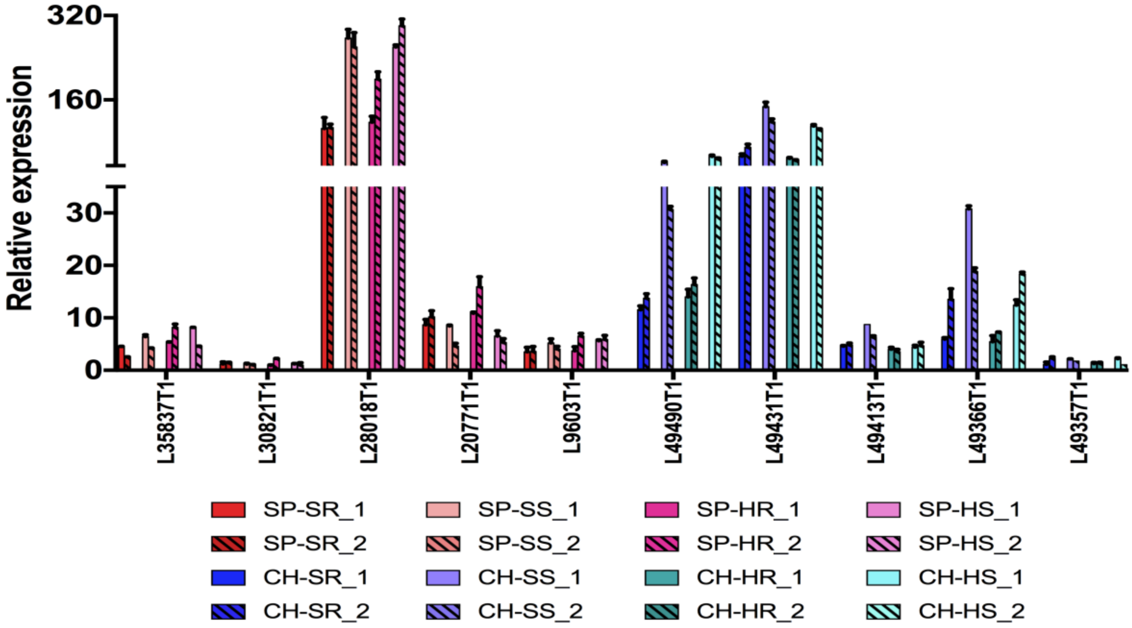

Figure S2. Pattern of expression of DETs between SP and CH in hydroponics roots. Results are presented as the ratio of the pattern of expression of DET between SP and CH, showing log2 values in the transcriptome libraries and the qRT-PCR analyses. For the expression in the transcriptome libraries, results are presented as the ratio SP/CH of the RPKM of each transcript. Bars represent mean ± standard deviation of the relative mRNA expression in two biological replicates. For the qRT-PCR data, results are presented as the ratio of expression of each transcript (relative to GAPDH and 28S rRNA) in each sample. Bars represent mean ± standard deviation of the relative mRNA expression in three biological replicates (3 plants) and each biological replicate analyzed with three technical replicates. .

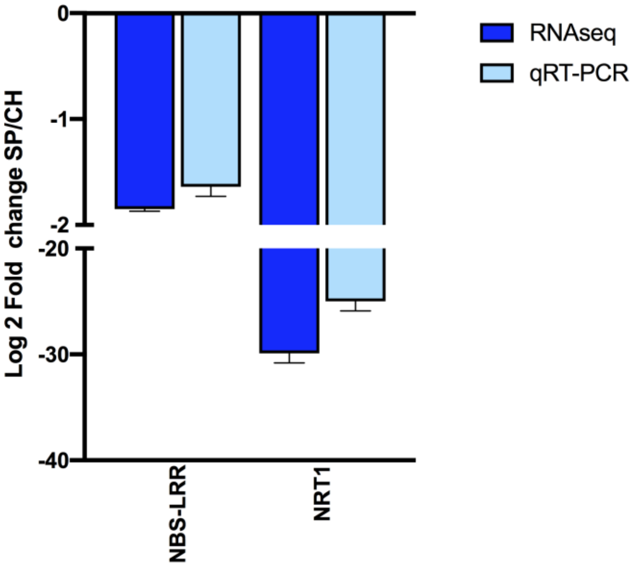

Figure S3. Exclusive transcripts identified in BNF contrasting genotypes. Patterns of expression of exclusive transcripts of SP or CH were evaluated by qRT-PCR in all 16 samples used in transcriptome sequencing analysis. The transcripts levels were normalized with plant rRNA and GAPDH in each sample. Each sample was representative of three plants, with three technical replicates and three biological replicates were evaluated.
